# Supplementary material for: Sex-Specific Differences in Fat Storage, Development of Non-Alcoholic Fatty Liver Disease and Brain Structure in Juvenile HFD-Induced Obese Ldlr-/-.Leiden Mice
Source: Nutrients. 2019 Aug 10;11(8):1861. doi: 10.3390/nu11081861 (PMC6723313; doi:10.3390/nu11081861)
Supplement: Supplementary file 1 [file nutrients-11-01861-s001.pdf]

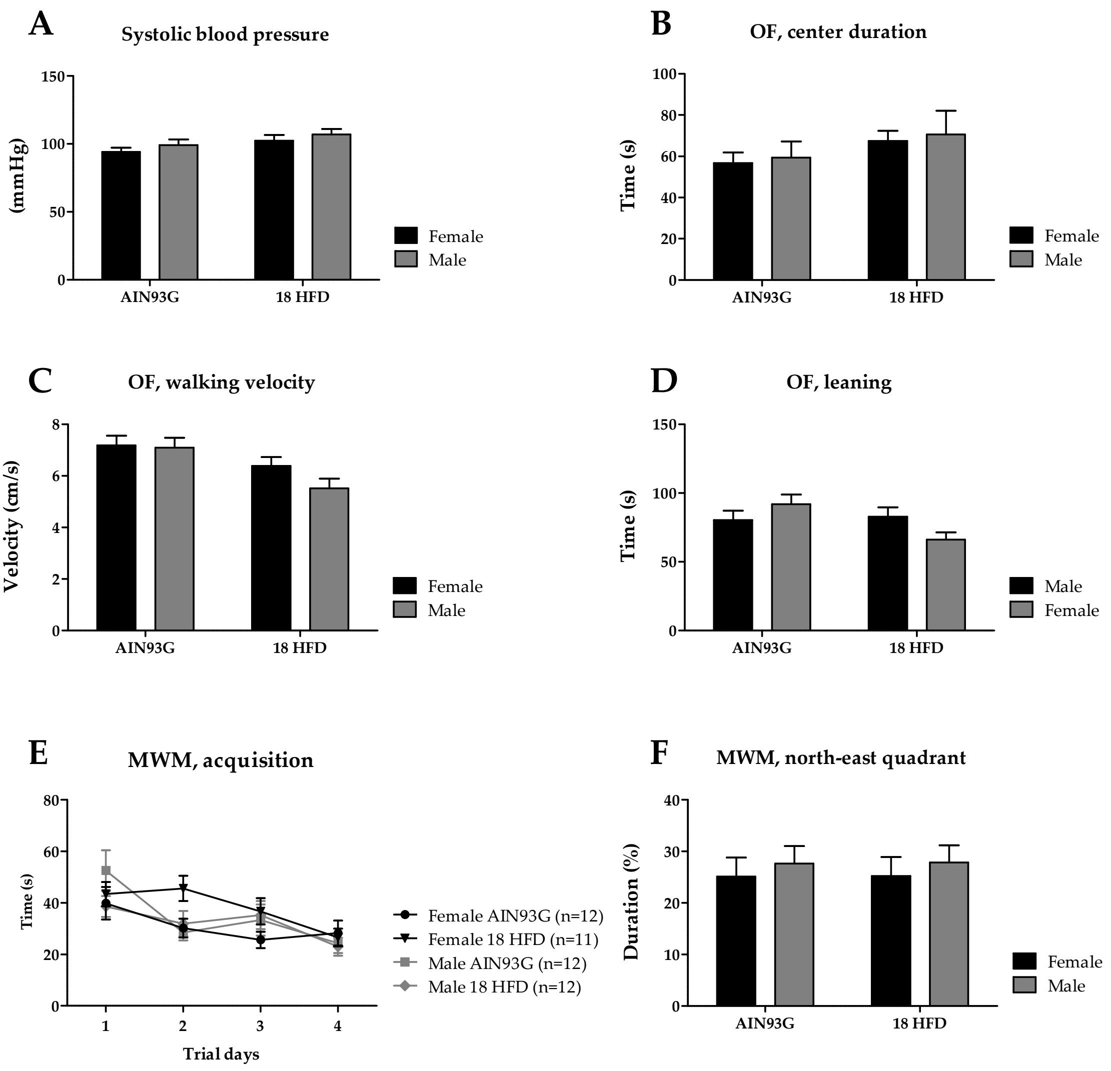

Figure S1. Analyses of systolic blood pressure, explorative behavior and spatial memory in young Ldlr-/-.Leiden male and female mice. (A) Systolic blood pressure measurement (mmHg). Parameters obtained in the open field test: (B) center duration, (C) walking velocity, and (D) wall leaning. E) Acquisition of the platform location in the Morris water maze (MWM) indicated spatial learning abilities. F) Spatial memory was indicated by percentage of time spend in the north-east quadrant (former platform location) during the probe-phase of the MWM.

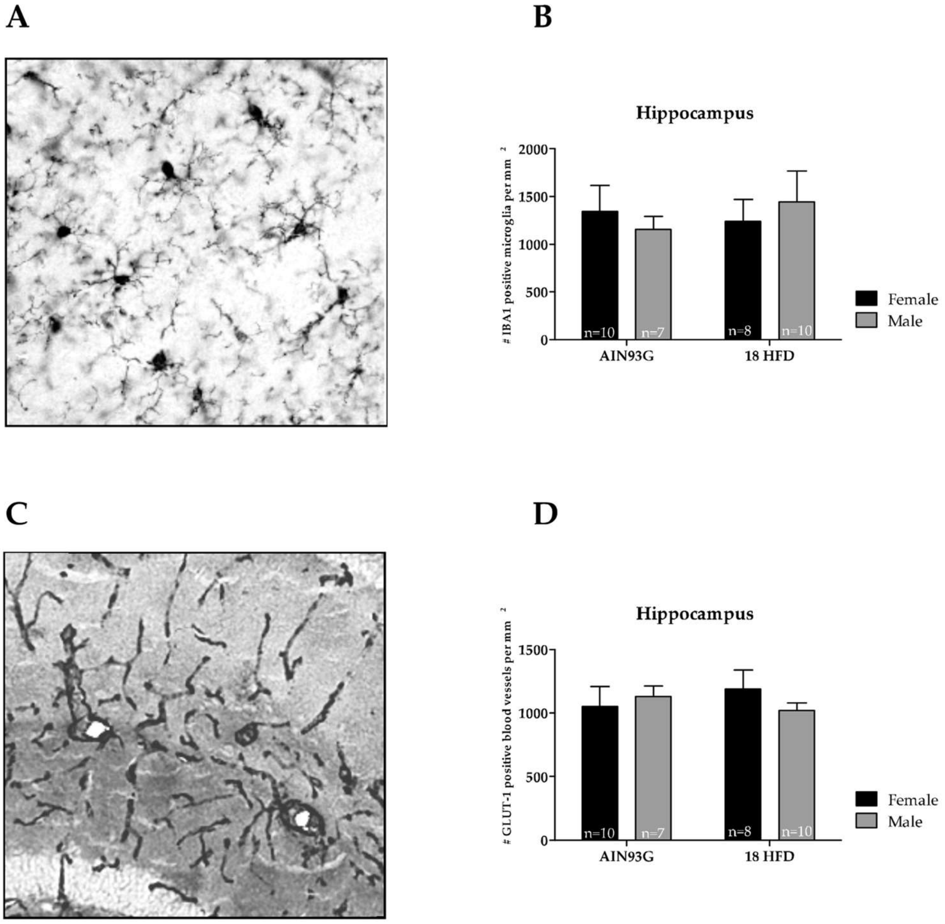

Figure S2. Analyses of neuroinflammation and cerebrovasculature in hippocampal sections of young Ldlr-/-.Leiden male and female mice. (A) Representative image of IBA-1 analyses in hippocampal sections (40x magnification). (B) Neuroinflammation indicated by the number of IBA-1 positive microglia per mm^2 in the hippocampus. (C) Representative image of GLUT-1 analysis in the hippocampus (5x magnification). (D) Quantification of GLUT-1 positive blood vessels (per mm^2) in the hippocampus. # number.
